# Evaluation of a Fully Automatic Measurement of Short-Term Variability of Repolarization on Intracardiac Electrograms in the Chronic Atrioventricular Block Dog

**DOI:** 10.3389/fphys.2020.01005

**Published:** 2020-08-21

**Authors:** Agnieszka Smoczyńska, David J. Sprenkeler, Alfonso Aranda, Jet D.M. Beekman, Alexandre Bossu, Albert Dunnink, Sofieke C. Wijers, Berthold Stegemann, Mathias Meine, Marc A. Vos

**Affiliations:** ^1^Department of Medical Physiology, University Medical Center Utrecht, Utrecht, Netherlands; ^2^Medtronic Bakken Research Center, Maastricht, Netherlands; ^3^Department of Cardiology, University Medical Center Utrecht, Utrecht, Netherlands

**Keywords:** ventricular arrhythmias, short-term variability of repolarization, automatic measurement, electrogram, activation recovery interval

## Abstract

**Background**: Short-term variability (STV) of repolarization of the monophasic action potential duration (MAPD) or activation recovery interval (ARI) on the intracardiac electrogram (EGM) increases abruptly prior to the occurrence of ventricular arrhythmias in the chronic AV-block (CAVB) dog model. Therefore, this parameter might be suitable for continuous monitoring of imminent arrhythmias using the EGM stored on an implanted device. However, 24/7 monitoring would require automatic STV_ARI_ measurement by the device.

**Objective**: To evaluate a newly developed automatic measurement of STV_ARI_ for prediction of dofetilide-induced torsade de pointes (TdP) arrhythmias in the CAVB-dog.

**Methods**: Two retrospective analyses were done on data from recently performed dog experiments. (1) In seven anesthetized CAVB-dogs, the new automatic STV_ARI_ method was compared with the gold standard STV_MAPD_ at baseline and after dofetilide administration (0.025 mg/kg in 5 min). (2) The predictive value of the automatic method was compared to currently used STV_ARI_ methods, i.e., slope method and fiducial segment averaging (FSA) method, in 11 inducible (≥3 TdP arrhythmias) and 10 non-inducible CAVB-dogs.

**Results**: (1) The automatic measurement of STV_ARI_ had good correlation with STV_MAPD_ (*r*^2^ = 0.89; *p* < 0.001). Bland-Altman analysis showed a small bias of 0.06 ms with limits of agreement between −0.63 and 0.76 ms. (2) STV_ARI_ of all three methods was significantly different between inducible and non-inducible dogs after dofetilide. The automatic method showed the highest predictive performance with an area under the ROC-curve of 0.93, compared to 0.85 and 0.87 of the slope and FSA methods, respectively. With a threshold of STV set at 1.69 ms, STV_ARI_ measured with the automatic method had a sensitivity of 0.91 and specificity of 0.90 in differentiating inducible from non-inducible subjects.

**Conclusion**: We developed a fully-automatic method for measurement of STV_ARI_ on the intracardiac EGM that can accurately predict the occurrence of ventricular arrhythmias in the CAVB-dog. Future integration of this method into implantable devices could provide the opportunity for 24/7 monitoring of arrhythmic risk.

## Introduction

Sudden cardiac arrest due to ventricular tachyarrhythmias, such as ventricular tachycardia (VT) or ventricular fibrillation (VF), is an important cause of death in patients with structural heart disease, accounting for approximately 50% of all cardiovascular deaths (Al-Khatib et al., 2018). Despite the widespread availability of automatic external defibrillators (AEDs), the prognosis after an out-of-hospital cardiac arrest remains poor, with an estimated survival rate around 10% (Benjamin et al., 2017). Therefore, focus has shifted toward preventive strategies in patients at high risk of sudden cardiac death (SCD).

Multiple randomized controlled trials have shown a survival benefit of the implantable cardioverter-defibrillator (ICD) in the prevention of SCD in patients with ischemic or non-ischemic cardiomyopathy and a reduced left ventricular ejection fraction (LVEF) below 35% (Moss et al., 2002; Bardy et al., 2005). Since the publication of these landmark trials, both European and American guidelines recommend ICD implantation as a class I indication for these patients (Priori et al., 2015; Al-Khatib et al., 2018). Nevertheless, while the ICD is highly effective in the prevention of SCD by termination of sustained ventricular tachyarrhythmias, the device does not prevent the arrhythmia itself from occurring. Despite being potentially life-saving, ICD discharges have also shown to cause severe psychological distress, depression, and anxiety and can reduce the quality of life in ICD recipients (Schron et al., 2002; Bostwick and Sola, 2007). Moreover, recurrent ICD shocks increase the number of hospital admissions and reduce the lifespan of the generator. Therefore, adjunctive therapy such as radiofrequency ablation of the arrhythmogenic substrate or administration of anti-arrhythmic drugs is often necessary to reduce the shock burden (Reddy et al., 2007; Van Herendael et al., 2010). However, both these treatment modalities expose the patient to potential adverse effects. In an ideal situation, the implanted device would not only terminate an arrhythmia that is already occurring but can also monitor if an arrhythmia is imminent and initiate preventive therapy (e.g., temporarily altering pacing rate) before the arrhythmia starts. However, the question remains how the device could predict if an arrhythmia is upcoming.

The chronic complete AV-block (CAVB) dog model is an arrhythmogenic animal model that is often used to evaluate new anti-arrhythmic agents or to study the mechanisms of ventricular tachyarrhythmias, mainly torsade de pointes (TdP) arrhythmias, in the remodeled heart. In this model, it has been shown that beat-to-beat variation of the monophasic action potential duration (MAPD), quantified as short-term variability (STV), increases abruptly a couple of minutes prior to the occurrence of TdP, after these animals are challenged with a pro-arrhythmic drug (Thomsen et al., 2007). Interestingly, the increase in STV was not seen in dogs that did not develop TdP after the same pro-arrhythmic challenge. While STV seems a promising parameter in predicting upcoming arrhythmic events, the use of monophasic action potential (MAP) catheters is not feasible for 24/7 monitoring in a clinical setting. Recently, it has been shown that STV of the activation recovery interval (STV_ARI_) of the electrogram (EGM) derived from the right ventricular ICD lead can be used as surrogate for the MAPD and accurately reflects arrhythmic risk in the CAVB dog under both anesthetized and awake conditions (Wijers et al., 2018). This would imply that the EGMs stored on the device could be used in the prediction of upcoming arrhythmic events. In the aforementioned study, however, the measurement of STV_ARI_ was done offline by use of semi-automatic custom-made software. In order to integrate continuous STV_ARI_ calculation in an implantable device, an automatic measurement is required that determines STV_ARI_ precisely, consistently, and in real-time without manual correction.

In the present study, we describe a new automatic method for measuring STV_ARI_ and compare this method with the current methodologies used to measure STV. In addition, we evaluate the potential of this automatic method in identifying imminent TdP arrhythmias in the CAVB dog model.

## Materials and Methods

Animal handling was in accordance with the “Directive 2010/63/EU of the European Parliament and of the Council of 22 September 2010 on the protection of animals used for scientific purposes” and the Dutch law, laid down in the Experiments on Animals Act. The Animal Experiment Committee of the University of Utrecht approved all experiments.

The current study is a retrospective analysis of data obtained during animal experiments performed in our laboratory between 2014 and 2017 and can be divided into two parts. In part 1, three different methods of STV_ARI_ measurement, including the newly developed automatic method, are compared to the golden standard STV_MAPD_. In part 2, the predictive value of the automatic method in identifying dogs inducible to TdP arrhythmias is evaluated and compared to the methods of STV_ARI_ measurement currently used in our department.

### Animal Experiments

The standard experimental procedures have been described in detail previously (Dunnink et al., 2010, 2012). After premedication with methadone 0.5 mg/kg, acepromazine 0.5 mg/kg, and atropine 0.02 mg/kg i.m., general anesthesia was induced *via* pentobarbital sodium 25 mg/kg i.v., and maintained by isoflurane 1.5% in O_2_ and N_2_O. During the experiments, 10 surface-ECG leads were recorded. Under aseptic conditions, the femoral artery and vein were dissected and sheaths were inserted. In the initial experiment, complete AV-block was created by radiofrequency ablation of the proximal His bundle. In the dogs included in part 1, a screw-in lead was placed in the right ventricular apex (RVA) *via* the jugular vein and connected to an internal pacemaker (Medtronic, Maastricht, The Netherlands), which was implanted subcutaneously. Subsequently, these dogs were left to remodel for at least 3 weeks during RVA pacing at the lowest captured rate, in the ventricular demand mode of ventricular pacing, sensing, and inhibition of pacing output in response to a sensed ventricular event (VVI). In contrast, the dogs selected for part 2 remodeled for at least 3 weeks on idioventricular rhythm (IVR).

At CAVB, a second experiment was performed to test for TdP susceptibility. Under general anesthesia, left and right ventricular (LV and RV) MAP catheters (Hugo Sachs Elektronik, March, Germany) and/or a duo-decapolar EGM catheter (St. Jude Medical, St. Paul, MN, USA) were inserted and advanced to the apex to record LV and RV MAPD and/or unipolar EGMs. A reference electrode was inserted in a superficial vein of the right hind leg. The I_Kr_ blocker dofetilide (0.025 mg/kg i.v., in 5 min or until the first TdP) was administered to test for inducibility of TdP arrhythmias. TdP was defined as a run of five or more short-coupled (occurring before the end of the T-wave) ectopic beats, with polymorphic twisting of the QRS-axis. If a TdP did not terminate by itself, the dog was defibrillated with 100 J by use of an external defibrillator. When ≥3 TdP’s occurred in the first 10 min after the start of infusion, the dog was considered inducible. During baseline and dofetilide challenge, dogs had either IVR or were paced from the RVA by the RV MAP catheter with a cycle length of 1,000 ms.

### Part 1 – Comparison of STV_ARI_ With STV_MAPD_

For part 1, adult mongrel dogs were selected that had both a LV EGM catheter and a LV MAP catheter in place during the experiment and were challenged for TdP inducibility with dofetilide. Since the simultaneous use of both LV EGM and LV MAP catheters is not common practice in our laboratory, only seven dogs (weight 27 ± 3 kg, all females) were included. All these dogs remodeled during RVA pacing at the lowest captured rate (59 ± 9 bpm) and were tested for inducibility at VVI60.

### Part 2 – Predictive Value of STV_ARI_ Measurements

**Figure 1:**
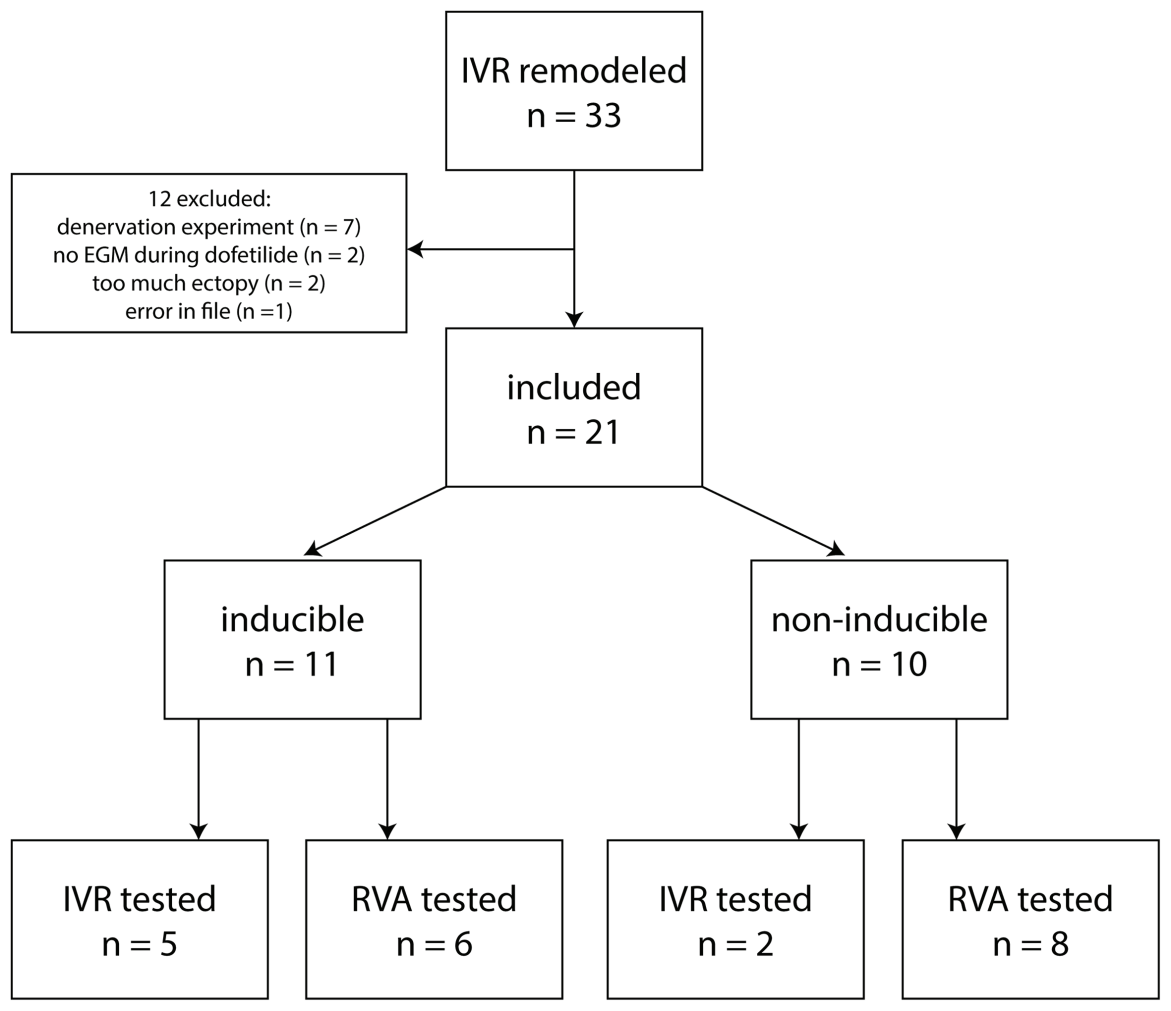
Flowchart of dogs included in part 2.

For part 2, dogs were selected that had remodeled on IVR, had an LV EGM catheter inserted, and were challenged for TdP inducibility with dofetilide. Of a total of 33 dogs in our database, 12 were excluded for various reasons (Figure 1), among which too much ectopy defined as the baseline recording of 10 min containing <2 consecutive minutes without ectopic beats. The 21 included dogs (weight 24.5 ± 3.2 kg, 7 males, 14 females) consisted of 11 inducible (5 tested at IVR, 6 at VVI60, 6 males) and 10 non-inducible dogs (2 tested at IVR, 8 at VVI60, 1 male).

### Data Analysis

**Figure 2:**
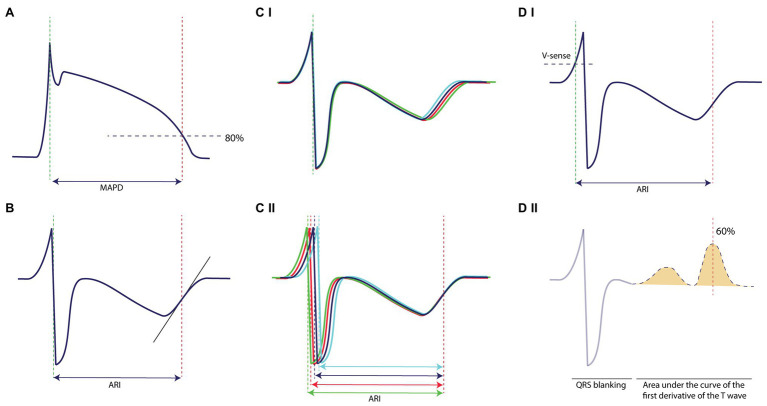
Methodology of measurement on the monophasic action potential and the electrogram. **(A)** STV_MAPD_ is measured from the initial peak until 80% of repolarization. **(B)** STV_ARI_ measured with the slope method from the minimum dV/dt of the QRS-complex to the maximum dV/dt of the T-wave, irrespectively of the morphology of the T-wave. **(C)** (I) STV_ARI_ measured with fiducial segment averaging from the minimum dV/dt of the QRS-complex to (II) the maximum dV/dt of the T-wave, which also form the fiducial points of alignment. **(D)** STV_ARI_ measured with the new automatic method, (I) whereby ventricular sensing by conventional device methodology indicates the start of the determinant of repolarization and (II) the T-wave end is determined as 60% of the area under the curve of the first derivative of the T-wave.

Both the surface ECG, LV MAPD, and LV unipolar EGM were recorded with EP Tracer (Cardiotek, Maastricht, The Netherlands) at a sampling frequency of 1,000 Hz. The RR-interval and QT-interval were measured offline with calipers on lead II. QT-interval was corrected for heart rate (QT_c_) with the van der Water formula (Van de Water et al., 1989). LV MAPD was measured semi-automatically from the initial peak until 80% of repolarization using custom-made software (AutoMAPD, MATLAB, MathWorks, Natick, MA, USA; Figure 2A). For comparison of STV_MAPD_ and STV_ARI_, we chose the EGM electrode that was located most closely to the MAP catheter under fluoroscopy. For calculation of STV_ARI_, the ARI of consecutive beats was determined by use of three different methods:

- Slope method (Figure 2B): ARI of every beat was measured from the minimum dV/dt of the QRS-complex to the maximum dV/dt of the T-wave, irrespectively of the morphology of the T-wave (either positive, negative, or biphasic).
- Fiducial segment averaging (FSA) method (Figure 2C): first, two fiducial points were defined, i.e., the minimum dV/dt of the QRS-complex as the ARI onset and the maximum dV/dt of the T-wave as the ARI offset. Using the method of FSA (Ritsema van Eck, 2002), all complexes were aligned separately around the ARI onset (Figure 2CI) and ARI offset (Figure 2CII) by cross-correlating the individual complex to the average of the other complexes until maximal correlation is achieved. The ARI of every single beat was then defined as the interval between the two fiducial points, taking the amount of shifting into account.
- Automatic method (Figure 2D): the obtained EGM signals were injected into an Evera ICD of Medtronic (sampling frequency 256 Hz) equipped with the algorithm to automatically determine STV in real-time. First, a complex was detected with the regular ventricular sensing method of the device (Figure 2DI). The QRS-complex was blanked to avoid interference in the T-wave end detection and was set at 140 ms (Figure 2DII). Once the QRS-complex was blanked, the first derivative of the resultant signal is calculated over time to detect changes in the slope. This gradient signal was then squared in order to make all data points positive and to emphasize slope changes in the signal (Figure 2DII). Finally, the T-wave end was defined as the point at 60% of the area under the curve of the resultant signal. The ARI is defined from the moment of ventricular sensing of the QRS-complex to the T-wave end, derived with this method (Figure 2DI).

Short-term variability of MAPD or ARI was calculated over 31 consecutive beats using the formula: $STV=\sum \left|D_{n+1}-D_{n}\right|/\left(30\times \sqrt{2}\right)$, where D represents MAPD or ARI. For STV calculation, ectopic beats and the first post-extrasystolic beat were excluded from analysis. All measurements were performed both at baseline and after administration of dofetilide. After dofetilide, parameters were determined just prior to the first ectopic beat. In the non-inducible dogs that did not show any ectopic beats, all parameters were assessed at the same time after start of dofetilide as measured in the inducible subjects.

### Statistical Analysis

Numerical values are expressed as mean ± standard deviation (SD). Comparison of serial data was performed with a paired Student’s *t*-test. Group comparison was done with an unpaired Student’s *t*-test. Group comparison with both a within-subject variable and a between-subject variable was performed with a mixed analysis of variance (ANOVA) with Sidak’s correction for multiple comparisons. The relation between the different STV modalities was analyzed by use of simple linear regression. In addition, Bland-Altman analysis was done to assess for systematic bias and limits of agreements. The area under the receiver operator characteristics (ROC) curve was used to evaluate the predictive power of the different STV_ARI_ methods. All statistical analyses were performed with GraphPad Prism 8.0 (GraphPad Software Inc., La Jolla, CA, USA). A value of *p* < 0.05 was considered as statistically significant.

## Results

### Part 1 – Comparison of STV_ARI_ With STV_MAPD_

Electrophysiological parameters at baseline and after dofetilide of the seven included animals are summarized in Table 1. From these seven animals, two were reproducibly inducible for TdP arrhythmias. All dogs were paced with a cycle length of 1,000 ms. As expected, dofetilide induced an increase in all repolarization parameters compared to baseline, including STV by all four different methods. MAPD and ARI_slope_ were comparable at baseline (251 ± 18 and 263 ± 21 ms, respectively, *p* = 0.26) and showed a similar increase after dofetilide (395 ± 53 and 396 ± 53 ms, respectively, *p* = 0.98). The highest values of STV, both at baseline and after dofetilide, were found when using the slope method (0.74 ± 0.26 and 2.67 ± 1.79 ms, respectively). The automatic method derived the lowest values of STV with a low SD (0.53 ± 0.24 ms at baseline and 1.52 ± 1.26 ms after dofetilide).

**Table 1:** Electrophysiological parameters of part 1 (*n* = 7).

	Baseline	Dofetilide
RR (ms)	1,000	1,000
QT (ms)	350 ± 20	499 ± 57^*^
QTc (ms)	350 ± 20	499 ± 57^*^
MAPD_80_ (ms)	251 ± 18	395 ± 53^*^
ARI (ms)	263 ± 21	396 ± 53^*^
STV_MAPD_ (ms)	0.58 ± 0.22	1.61 ± 1.37^*^
STV_ARI_ slope (ms)	0.74 ± 0.26	2.67 ± 1.79^*^
STV_ARI_ FSA (ms)	0.61 ± 0.21	1.97 ± 1.06^*^
STV_ARI_ automatic (ms)	0.53 ± 0.24	1.52 ± 1.26^*^

Data expressed as mean ± SD in ms.

* *p* < 0.05 compared to baseline.

**Figure 3:**
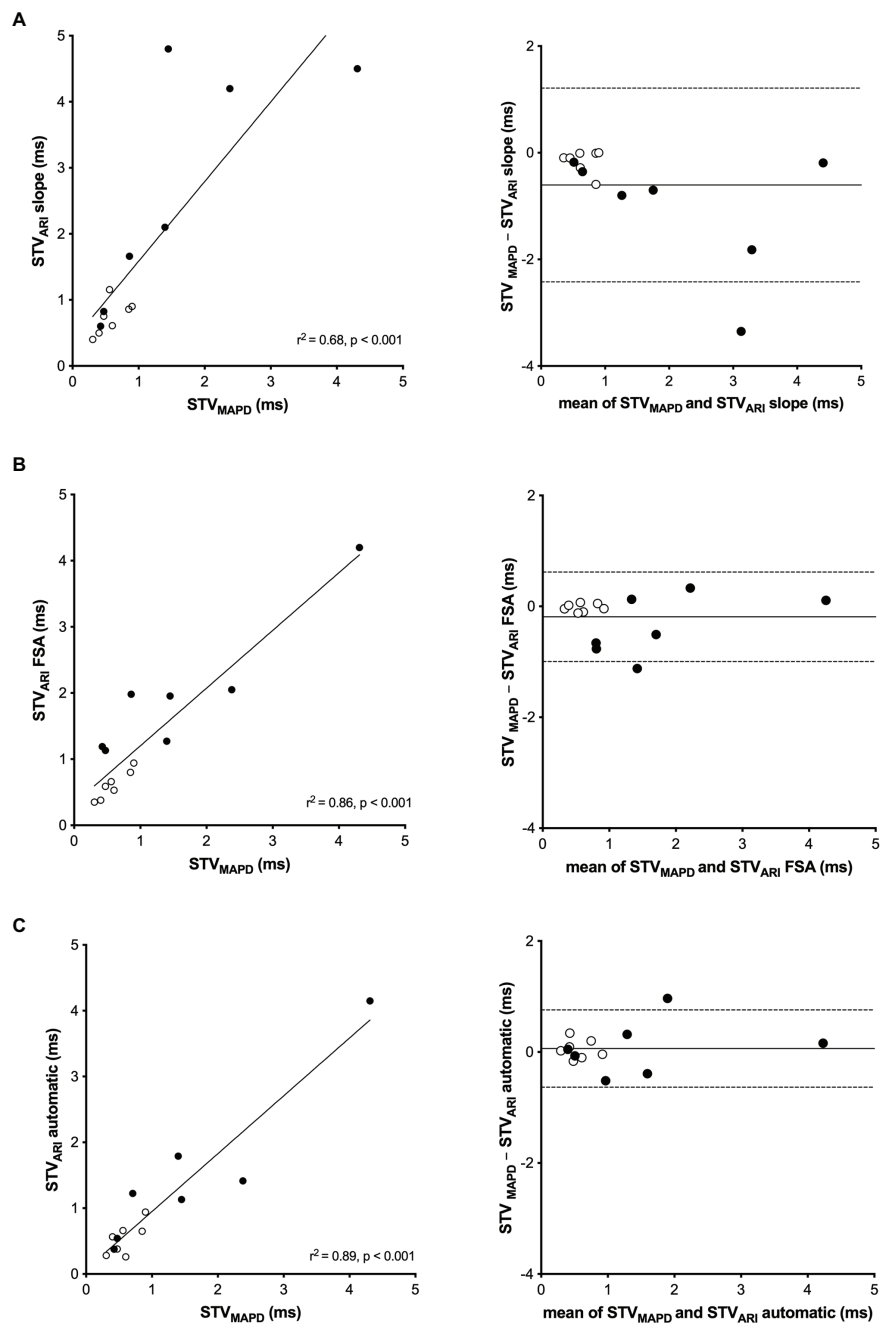
Linear regression and Bland-Altman analysis of STV_ARI_ compared to STV_MAPD_. **(A)** STV_MAPD_ vs. STV_ARI_ measured with the slope method. **(B)** STV_MAPD_ vs. STV_ARI_ measured with the fiducial segment averaging (FSA) method. **(C)** STV_MAPD_ vs. STV_ARI_ measured with the new automatic method.

Figure 3 shows the regression analysis and Bland-Altman plots of the three different STV_ARI_ methods compared to the gold standard STV_MAPD_. The slope method had a moderate correlation with STV_MAPD_ (*r* ^2^ of 0.68, *p* < 0.001). A systematic bias was found of −0.61 ms with increasing differences between the two methods at higher values. The limits of agreement of the Bland-Altman plot were −2.42 and 1.21 ms. The FSA method had a better correlation with an *r* ^2^ of 0.86 (*p* < 0.001). A small bias of −0.20 was found with limits of agreement between −0.99 and 0.62 ms. The new automatic method showed a very good correlation with STV_MAPD_ with an *r*^2^ of 0.89 (*p* < 0.001). A negligibly small systematic bias was seen of 0.06 ms with a narrow bandwidth of agreement between −0.63 and 0.76 ms.

### Part 2 – Predictive Value of STV_ARI_ Measurements

**Figure 4:**
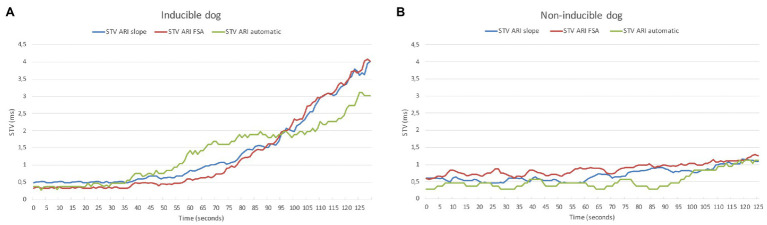
Example of STV_ARI_ in inducible **(A)** and non-inducible dog **(B)** after dofetilide. STV_ARI_ measured with the slope method (blue line), fiducial segment averaging (FSA) method (red line), and automatic method (green line) from the moment of dofetilide infusion at time = 0 s.

In Table 2, the electrophysiological parameters at baseline and after dofetilide of the 21 included dogs are shown, separately analyzed for inducible and non-inducible dogs. No differences were found in RR-interval, QT-interval, QTc-interval, or ARI between inducible and non-inducible dogs, both at baseline and after administration of dofetilide. In addition, at baseline, STV_ARI_ was similar for inducible and non-inducible dogs. After administration of dofetilide, the STV_ARI_ measured by all different methods increased significantly in the inducible dogs, creating a significant difference in STV_ARI_ between inducible and non-inducible subjects after dofetilide. Figure 4 shows an example of the effect of dofetilide infusion on the three different methods of STV_ARI_ in both inducible and non-inducible dogs. While the non-inducible dog shows only a mild increase of STV_ARI_, a prominent rise in STV is observed in the inducible dog prior to the occurrence of TdP arrhythmias.

**Table 2:** Electrophysiological parameters of part 2 (*n* = 21).

	Baseline			Dofetilide		
	Total (*n* = 21)	I (*n* = 11)	NI (*n* = 10)	Total (*n* = 21)	I (*n* = 11)	NI (*n* = 10)
RR (ms)	1,186 ± 255	1,263 ± 229	1,102 ± 267	1,197 ± 257	1,274 ± 219	1,111 ± 266
QT (ms)	395 ± 57	403 ± 59	387 ± 56	569 ± 72^*^	578 ± 76^*^	560 ± 75^*^
QTc (ms)	379 ± 59	380 ± 61	378 ± 59	522 ± 70^*^	554 ± 70^*^	550 ± 71^*^
ARI (ms)	296 ± 49	309 ± 52	282 ± 44	411 ± 88^*^	443 ± 85^*^	375 ± 80^*^
STV_ARI_ slope (ms)	1.44 ± 1.04	1.69 ± 1.33	1.15 ± 0.49	3.57 ± 2.75^*^	5.09 ± 3.07^*^	1.90 ± 0.64^§^
STV_ARI_ FSA (ms)	1.14 ± 0.68	1.20 ± 0.88	1.08 ± 0.42	2.41 ± 1.25^*^	3.11 ± 1.30^*^	1.63 ± 0.57^§^
STV_ARI_ automatic (ms)	1.00 ± 0.58	1.02 ± 0.63	0.98 ± 0.54	1.93 ± 0.95^*^	2.51 ± 0.96^*^	1.30 ± 0.33^§^

Data expressed as mean ± SD in ms.

* *p* < 0.05 compared to baseline.

§ *p* < 0.05 compared to inducible subject after dofetilide administration.

**Figure 5:**
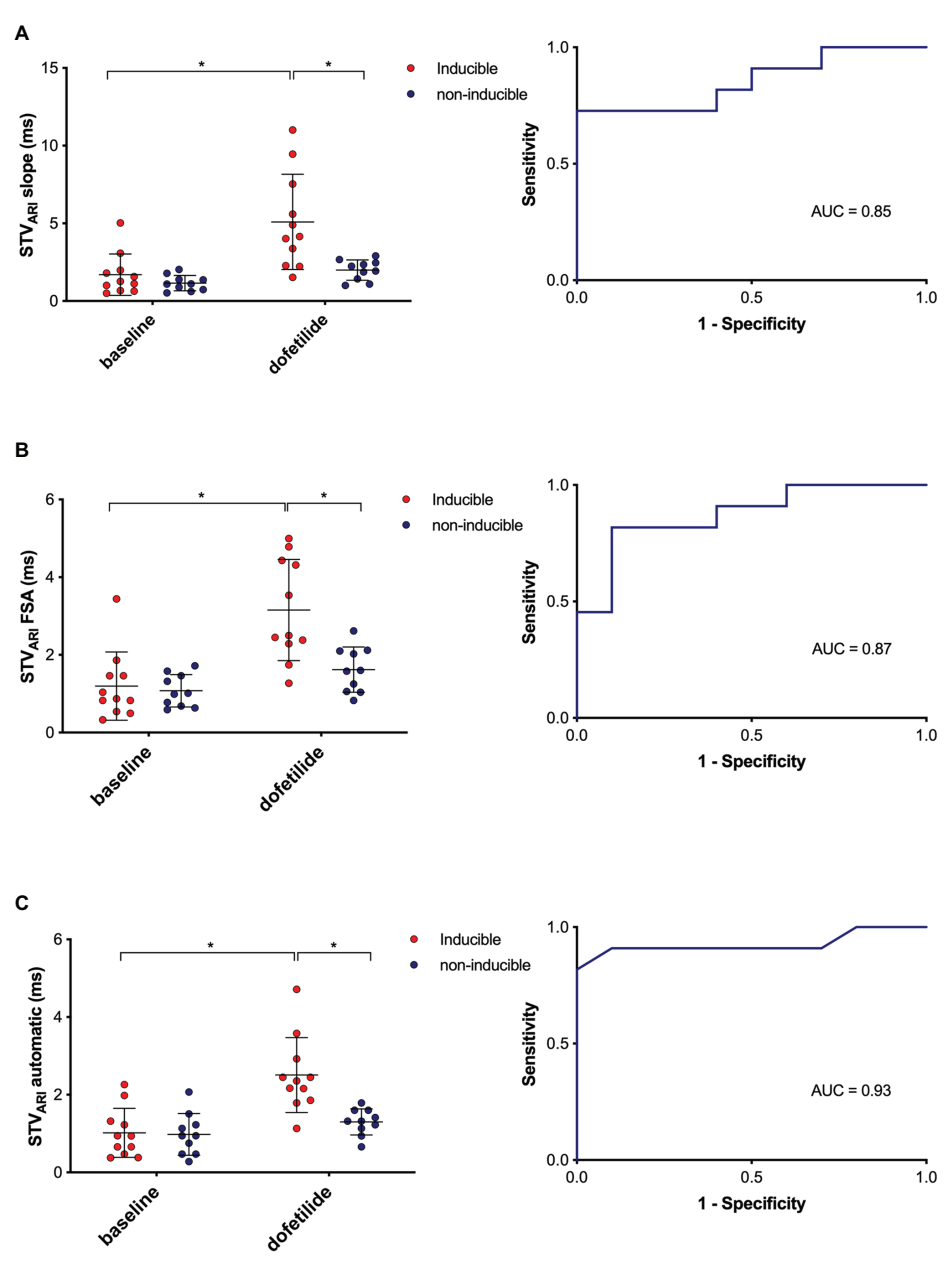
Predictive value of different methods of STV_ARI_. STV_ARI_ at baseline and after dofetilide in inducible (*red*) and non-inducible dogs (*blue*) measured with the slope method **(A)**, FSA method **(B),** and automatic method **(C)** with the corresponding receiver operating characteristics (ROC) curves. AUC, area under the curve. *p < 0.05.

The predictive performance of the three methods was evaluated, i.e., to what extent the different methods of STV_ARI_ were able to distinguish between dogs that were inducible versus those that were not inducible to drug-induced TdP arrhythmias. In Figure 5, the STV_ARI_ of the three methods is presented separately for inducible and non-inducible dogs, both at baseline and dofetilide. One can clearly see that, while STV_ARI_ measured with the slope and FSA method partly overlap for some inducible and non-inducible subjects after dofetilide, an almost total separation in STV_ARI_ between the two groups can be found with the new automatic measurement. This is further illustrated in the ROC-curves of the different methods. The automatic method results in the highest area under the curve (AUC) of 0.93 (95% CI 0.79–1.00), compared to an AUC of 0.85 (95% CI 0.69–1.00) and 0.87 (95% CI 0.72–1.00) of the slope method and FSA method, respectively. With a threshold set at 1.69 ms, the automatic method yields a sensitivity of 0.91 (95% CI 0.59–0.99) and a specificity of 0.90 (95% CI 0.55–0.99).

## Discussion

In the present study, we evaluated a fully automatic method of STV_ARI_ measurement in the CAVB dog model and compared this new method with the current gold standard STV_MAPD_ and two other methods used in our laboratory to derive STV_ARI_ from the intracardiac EGM. The results show that (1) STV_ARI_ determined with the automatic method is highly comparable to STV_MAPD_ both under baseline conditions and after dofetilide, (2) the automatically determined STV_ARI_ performs better in predicting imminent TdP arrhythmias after dofetilide administration compared to the current STV_ARI_ methods.

### STV as a Marker of Arrhythmic Risk

As expected, of all electrophysiological parameters, only STV could distinguish between inducible and non-inducible subjects after dofetilide administration (Table 2). Previous studies in the CAVB dog model have well established STV as a better marker of reduced repolarization reserve and arrhythmic risk compared to parameters of repolarization duration (QT, MAPD, or ARI) alone (Thomsen et al., 2004, 2006b). In pro-arrhythmic drug safety testing, STV was able to identify safe from unsafe drugs despite similar QT-prolongation (Thomsen et al., 2006a; Varkevisser et al., 2012). In addition, reduction of STV is highly related to efficacy of anti-arrhythmic agents (Bossu et al., 2017). Furthermore, a dynamic behavior of STV prior to the occurrence of arrhythmias has been demonstrated. Thomsen et al. (2007) observed that after infusion of dofetilide, STV of LV MAPD shows a steep increase just prior to the occurrence of TdP-arrhythmias, which was not present in the non-inducible dogs. The extent of the increase in STV has also been correlated to the severity of the arrhythmic outcome. A more severe arrhythmic outcome requiring >3 defibrillations during the 10 min following dofetilide infusion was preceded by a higher increase in STV of LV MAPD than a moderate arrhythmic outcome consisting of only self-terminating TdP-arrhythmias (Smoczyńska et al., 2019). Therefore, STV dynamics could provide important information about impending arrhythmic events. Since MAP-catheters are rarely used outside the experimental laboratory, recent interest shifted toward measurement of STV on the EGM of chronically implanted ICD-leads (Oosterhoff et al., 2010, 2011; Wijers et al., 2018). These leads have the advantage of recording EGMs 24/7 from a fixed position on the myocardium.

### Different Methods of STV_ARI_ Compared to STV_MAPD_

We demonstrated a moderate to good correlation between STV_ARI_ of the LV EGM with STV of the LV MAPD, with an *r*^2^ ranging from 0.68 to 0.89 for the different methodologies (Figure 3). This finding is in line with results of previous studies. Oosterhoff et al. (2010) compared STV LV MAPD and STV LV ARI measured *via* an epicardial inserted screw electrode with a slightly lower sampling rate of 800 Hz. Despite the methodological differences, a similar correlation between STV ARI and STV MAPD was found with an *r*^2^ of 0.71. Recently, Wijers et al. (2018) observed a positive correlation between STV MAPD and STV ARI from the RV EGM. Yet, the correlation was less strong (*r*^2^ = 0.41), possibly due to differences in sampling frequency between the MAP and EGM recording (1,000 Hz for the MAP and 250 Hz, resampled to 400 Hz, for the EGM).

When comparing the three methods of STV_ARI_, important differences in correlation with STV_MAPD_ are found. The slope method had the weakest correlation with an *r*^2^ of 0.68. This method incorporates the widely used definition of ARI as proposed by Wyatt (1980), who showed that the interval from the minimum dV/dt of the QRS-complex to the maximum dV/dt of the T-wave of the unipolar EGM correlates highly with action potential duration. While there is general consensus on the use of the minimum dV/dt as local depolarization time, a debate exists on whether the maximum or minimum dV/dt of the T-wave should be used as index of local repolarization time, especially when the T-wave is positive or biphasic. However, recent computer simulation and experimental studies have demonstrated that, irrespective of T-wave morphology, the upstroke of the T-wave coincides with repolarization on the MAP and that the minimum dV/dt of a positive T-wave represents remote repolarization (Coronel et al., 2006; Western et al., 2015). Therefore, we have chosen to use maximal upstroke as index of local repolarization for all T-wave morphologies (negative, positive, or biphasic). Nevertheless, most of the EGMs used for the analysis had negative upsloping T-waves, possibly due to their location in the apex of the heart. Therefore, the definition of ARI offset does not seem the reason for the weak correlation of the slope method with MAPD. While at baseline STV_ARI_ by the slope method is more or less similar to STV_MAPD_, the discrepancy between the two methods starts to arise after dofetilide (Figure 3A). This is further illustrated by the Bland-Altman plot, which shows that the systematic bias of the slope method increases at higher average values. The explanation for this bias is probably related to the inaccuracy of determining maximal dV/dt of the T-wave at longer ARIs. After infusion of dofetilide, the ARI prolongs and the T-wave widens. Hence, the upstroke of the T-wave becomes less steep, making it difficult to determine the exact point of maximal dV/dt for every beat, which introduces measurement error. Since the STV formula uses absolute differences between consecutive ARI, there is no cancellation of measurement error, resulting in a higher STV.

STV_ARI_ measured with the FSA method shows better correlation with STV_MAPD_ and has no clear systematic bias on the Bland-Altman plot. FSA has been developed to accurately determine intervals between certain fiducial points and thus prevents the aforementioned problem of summation of measurement error that is caused by determining ARI for every single beat separately. FSA determines the fiducial points (i.e., ARI onset and the ARI offset) for all complexes at once. After alignment of the complexes around these fiducial points, the individual shifts between the fiducial points are used to calculate the individual ARIs. Therefore, the beat-to-beat variations in ARI are preserved, without introducing repeated measurement error. However, while this method shows improved accuracy in measurement of STV compared to the slope method, the use of FSA for continuous monitoring in an implanted device is problematic, because it requires a lot of computing memory for the calculation to be performed. Therefore, we tried to come up with a new method that is both easy to calculate and will not drain the battery of the device, even when used continuously.

The automatic method of STV_ARI_ shows an excellent correlation with STV_MAPD_ with an *r*^2^ of 0.89 despite the lower sampling frequency of 256 Hz in the device compared to the MAP signal of 1,000 Hz. Oosterhoff et al. (2010) investigated the effect of sampling frequency on measurement of STV of the ARI and MAPD by using computer simulations. The lowest sampling frequency of 250 Hz had the highest SD at low values of STV compared to a higher sampling frequency of 1,000 Hz. However, in the current study, the automatic method of STV_ARI_ yielded lower or similar SDs at the sampling frequency of 250 Hz as the other methodologies at a sampling frequency of 1,000 Hz. The automatic method may be more robust and able to perform well at a lower sampling frequency, because it determines the T-wave end based on the area under the slope of the first derivative of the signal, rather than one exact point in the original signal of the T-wave.

### The Performance of STV_ARI_ in Prediction of TdP Arrhythmia

In part 2 of the study, the three different methods were evaluated on their ability to identify a change in STV_ARI_ prior to the occurrence of TdP arrhythmias in the inducible dogs. At baseline, all electrophysiological parameters including STV_ARI_ were similar for inducible and non-inducible dogs (Table 2). This is in contrast to the study by Thomsen et al. (2007), which already found a difference in STV_MAPD_ at baseline between inducible and non-inducible subjects. However, since the values of STV are very low at baseline, it is imaginable that you do not find a difference here. Nonetheless, after infusion of dofetilide, all three STV methods showed a significant higher STV_ARI_ in the inducible dogs prior to the occurrence of first ectopic beat compared to a similar timepoint in the non-inducible dogs. While the slope method derived the largest absolute difference of STV_ARI_ between inducible and non-inducible dogs (5.09 vs. 1.90 ms), there was also a high variability, with some dogs showing a very large increase in STV, while others had STV values comparable to the non-inducible subjects. This resulted in a lower predictive capability as seen by a lower AUC of the ROC curve. The same applies for the FSA method. On the other hand, the new automatic method could almost completely separate inducible from non-inducible dogs, resulting in a very high specificity and sensitivity. This would make it possible to define a specific threshold, above which STV_ARI_ predicts, with high accuracy, the occurrence of upcoming arrhythmic events.

### Clinical Utility of the Intracardiac EGM

A number of clinical studies have evaluated the use of intracardiac EGMs for the prediction of ventricular tachyarrhythmias. Tereshchenko et al. (2009) analyzed the predictive value of QT variability index (QTVI) on intracardiac EGMs in 298 ICD-patients. The highest quartile of QTVI was an independent predictor of VT/VF and appropriate ICD therapy at a mean follow-up of 16 months. In addition, Sandhu et al. (2008) analyzed intracardiac T-wave alternans on the EGM during an electrophysiological study in 78 patients and found a positive predictive value of only 14% but a high negative predictive value of 95% at 1 year. However, both these studies investigated the use of intracardiac EGM parameters for prediction of sustained arrhythmias during long-term follow-up. Swerdlow et al. (2011) did a prospective multicenter study in 68 ICD-patients and evaluated T-wave alternans and non-alternans T-wave variability (TWA/V) on EGMs preceding spontaneous ventricular tachyarrhythmias. They observed a significantly higher TWA/V immediately before the occurrence of arrhythmias compared to four control recordings. In line with our findings, this study demonstrates that the intracardiac EGM can provide valuable information for the prediction of imminent life-threatening arrhythmias in a clinical setting. However, this information would only be useful if appropriate treatment, such as alternative pacing algorithms, can be initiated in time to prevent the arrhythmia from occurring. Recently, Wijers et al. (2017) demonstrated that temporary accelerated pacing (TAP) initiated just after the first ectopic beat can prevent TdP-arrhythmias in the CAVB-dog. This is in agreement with clinical studies that show that rate-smoothing pacing algorithms can reduce the number of sustained ventricular tachyarrhythmias in ICD-recipients and patients with long QT-syndrome (Viskin et al., 2000; Wietholt et al., 2003). Therefore, by use of 24/7 automatic measurement of STV_ARI_ in real-time, the device does not have to wait for an ectopic beat to initiate pacing therapy but can already start with treatment when STV rises above a certain threshold value, thereby preventing the arrhythmia and ICD shock.

### Limitations

Certain limitations of the current study must be addressed. First, this study had a retrospective study design, therefore, not all variables could be controlled. This resulted in important differences between the dogs used for the analyses of part 1 and part 2. For the comparison of STV_MAPD_ and STV_ARI_ in part 1, we were limited in the number of animals, because the combined use of an LV MAP and LV EGM catheter has only been done in a few experiments, in which dogs remodeled at low rate RVA pacing. It is known from previous (unpublished) data from our laboratory that control of the activation pattern by pacing can influence the course of electrical remodeling and the arrhythmic susceptibility compared to dogs that remodeled on their own IVR. Therefore, for part 2, in which no additional MAP catheter was required, we selected only IVR remodeled dogs to maintain a more homogeneous population. It should also be noted that we included both dogs that had IVR or were RVA paced at VVI60 during the experiment. Since the IVR can be lower than 60/min, these dogs could have longer APD and thus higher STV, which may act as a possible confounder in the differences of STV between inducible and non-inducible dogs. Finally, we should acknowledge that the CAVB dog is a specific animal model sensitive to triggered-activity based TdP arrhythmias. Therefore, extrapolation of the predictive value of STV to other types of ventricular tachyarrhythmias should be done with caution.

### Future Directions

The development of a reliable and accurate automatic method of STV measurement on intracardiac EGMs is an important step toward clinical implementation of STV for continuous monitoring. Prospective observational studies in ICD patients should evaluate if the same increase in STV_ARI_ prior to the occurrence ventricular arrhythmias is observed in a clinical setting. Furthermore, in future studies, the detection mode of predicting imminent arrhythmias should be coupled to a fully automatic algorithm for the initiation of preventive pacing therapy, such as TAP. When a sudden increase of STV above a certain threshold value is detected, the device could then automatically start a pacing algorithm, thereby preventing the arrhythmia from occurring.

### Conclusion

In conclusion, we have developed a new fully-automatic method of measuring STV_ARI_ on the intracardiac EGM in the CAVB dog model. This method is highly correlated to the gold standard STV_MAPD_ and can accurately identify a pro-arrhythmic rise in STV_ARI_ prior to the occurrence of dofetilide induced TdP-arrhythmias. This technique could be integrated in implantable devices for prediction of imminent ventricular tachyarrhythmias and initiation of preventive pacing therapy.

## Data Availability Statement

The raw data supporting the conclusions of this article will be made available by the authors, without undue reservation.

## Ethics Statement

The animal study was reviewed and approved by The Animal Experiment Committee of the University of Utrecht.

## Author Contributions

AS and DS analyzed data and wrote the manuscript. AA developed the automatic algorithm. DS, JB, AB, AD, and SW did experimental work. BS and MM contributed to experimental concepts and design of the study. MV was responsible for organization of experiments and revised the manuscript thoroughly. All authors contributed to the article and approved the submitted version.

### Conflict of Interest

AA and BS were employed by the Bakken Research Center in Maastricht of Medtronic.

The remaining authors declare that the research was conducted in the absence of any commercial or financial relationships that could be construed as a potential conflict of interest.
